# “Eggs in egg cartons”: co-crystallization to embed molecular cages into crystalline lattices†

**DOI:** 10.1039/d0sc03191g

**Published:** 2020-09-15

**Authors:** Yuya Domoto, Masahiro Abe, Kidai Yamamoto, Takashi Kikuchi, Makoto Fujita

**Affiliations:** Department of Applied Chemistry, The University of Tokyo 7-3-1 Hongo, Bunkyo-ku Tokyo 113-8656 Japan domoto@appchem.t.u-tokyo.ac.jp mfujita@appchem.t.u-tokyo.ac.jp; Rigaku Corporation 3-9-12 Matsubara-cho Akishima-shi Tokyo 196-8666 Japan; Division of Advanced Molecular Science, Institute for Molecular Science, National Institutes of Natural Sciences 5-1 Higashiyama, Myodaiji-cho Okazaki-shi Aichi 444-8787 Japan

## Abstract

Discrete (M_3_L_2_)_*n*_ cages assembled from a tripodal ligand (L) and metal ions (M: Cu(i) or Ag(i)) are embedded in networked coordination hosts formed by partial dissociation of the same discrete cages during the crystallization process. The resulting “eggs-in-an-egg-carton” structures provide unique examples of the co-crystallization of discrete and infinite coordination frameworks.

## Abstract 2

Discrete coordination cages were connected into the infinite lattices *via* shape-complementary co-crystallization with networked coordination hosts in the “eggs-in-an-egg-carton” styles.

## Graphical abstract

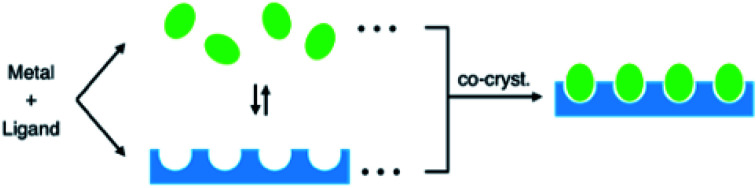

## Introduction

**Fig. 1:**
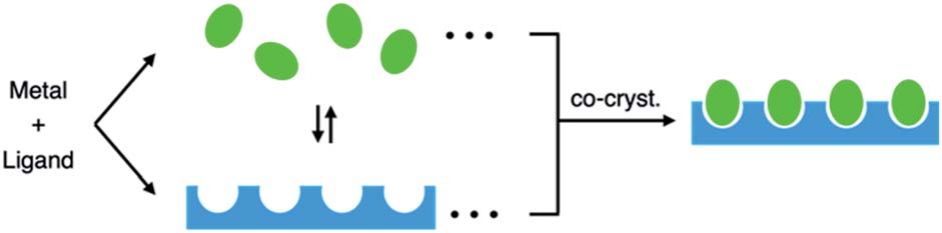
Cartoon representation of discrete–infinite (eggs-in-an-egg-carton) co-crystallization.

The connection of discrete cages into infinite chemical structures is an attractive strategy for translating the properties and functions of cages from solution into the solid state.^1,2^ The simplest method of discrete-to-infinite translation is polymerization of the cage units, and this has been achieved for self-assembled coordination and covalent cages by (i) sharing metal centres among adjacent cages,^2*a*,*b*^ (ii) linking cage units with polydentate counter ions or additional bridging ligands,^2*c*,*f*,3*b*,*c*^ or (iii) forming dynamic covalent bonds at peripheral reaction sites on the cages.^3*d*^ However, in all of these methods, post-modification of the original cage is necessary. Here, we report the formation of crystalline materials with predetermined cavities and a structure reminiscent of eggs in an egg carton through co-crystallization of discrete coordination cages with infinite coordination lattices (Fig. 1). The two components, the discrete cages (eggs) and the infinite lattice (egg carton), need not be prepared independently because the latter is formed by partial dissociation of the original discrete cages during the crystallization process.

## Results and discussion

**Fig. 2:**
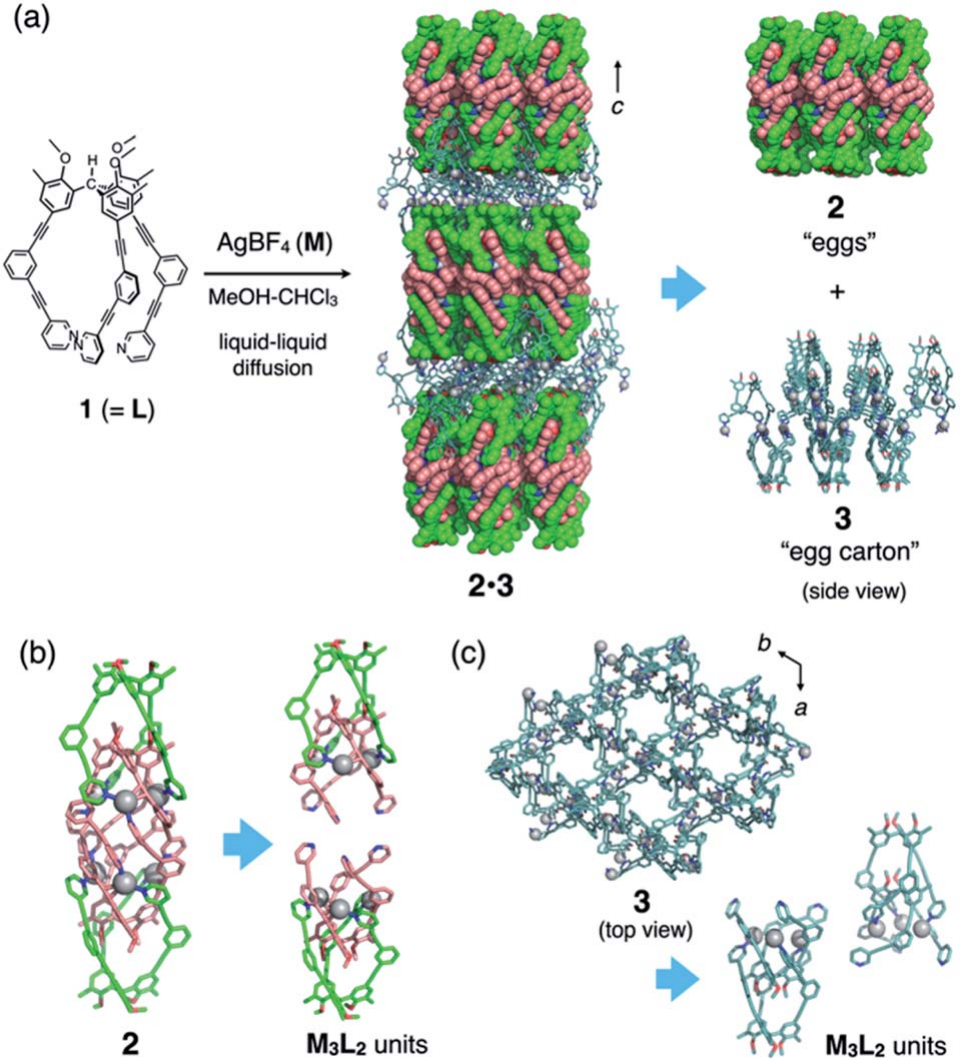
Synthesis and structure of “eggs-in-an-egg-carton” complex **2·3**. (a) Synthetic scheme and crystal structure of **2·3**, which consists of discrete cages **2** and infinite framework **3**. (b) Interlocked cage structure of **2**, composed of two M_3_L_2_ units. Encapsulated two BF_4_^−^ ions are omitted for clarity. (c) Infinite framework of **3** (top view), composed of the same M_3_L_2_ units.

**Fig. 3:**
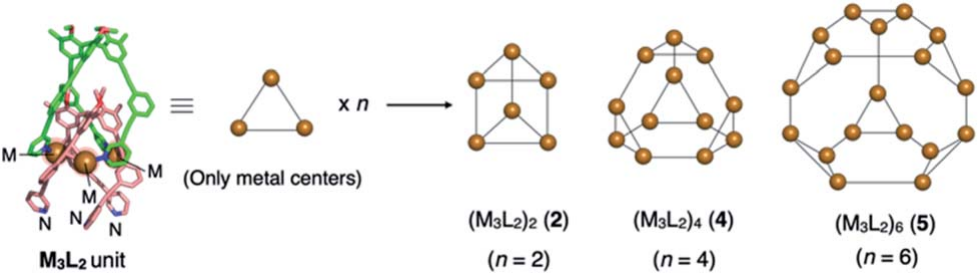
Cartoon representation of the (M_3_L_2_)_*n*_ oligomeric cages. The three metal centres of the common M_3_L_2_ subunit are extracted from each crystal structure. Depending on the assembly conditions, this subunit oligomerizes into dimeric, tetrameric, and hexameric cages (**2**, **4**, and **5**, respectively) by coordination of the free pyridyl nitrogen atoms of one subunit to the vacant metal centres (M) of the other subunits. For the complete structures, see the ESI† or our previous report.^4^

We recently reported the self-assembly of highly inter-penetrated discrete cages (M_3_L_2_)_*n*_ (*n* = 2, 4, 6)^4^ from tripodal ligand **1** (Fig. 2a) and metal ions (Cu(i) or Ag(i)). The acetylene spacers in ligand **1** play a key role in the formation of the complex framework; the weak metal–acetylene interaction^5,6^ works concertedly with the relatively strong metal–pyridyl coordination. All structures contain the common M_3_L_2_ subunit, which formally oligomerizes into dimeric, tetrameric, or hexameric cages depending on the assembly conditions (Fig. 3).

We now find that this common subunit can also undergo oligomerization into an infinite framework during crystallization of the discrete (M_3_L_2_)_*n*_ cages. As a result, we obtained discrete–infinite co-crystallized structures in which discrete cages were embedded in an (M_3_L_2_)_*n*_ infinite lattice with a structure reminiscent of eggs in an egg carton (Fig. 1).

The discrete–infinite co-crystal was obtained during our attempts to obtain a single crystal of the (M_3_L_2_)_6_ hexameric cage (Fig. 3) by using a liquid–liquid diffusion method. A solution of AgBF_4_ (7.9 mM in MeOH, 1.5 eq.) was layered on a solution of ligand **1** (5.2 mM in CHCl_3_) in a glass tube. After maintaining the layered solution at 30 °C for 2 weeks, single crystals formed in the tube with moderate yield (56%). Single-crystal X-ray analysis of these crystals revealed the uncommon discrete–infinite co-crystal **2·3** ([(Ag_3_(**1**)_2_)_2_(BF_4_)_6_][Ag_3_(**1**)_2_(BF_4_)_3_](solv)_*n*_), in which two composites, discrete (M_3_L_2_)_2_ cage **2** (with dimensions of *ca.* 3.8 × 1.4 × 1.4 nm) and (M_3_L_2_)_*n*_-type infinite framework **3**, co-exist (Fig. 2a). As shown in Fig. 2b and c, both composites contain the common M_3_L_2_ subunit, with the comparable average N–Ag distances between **2** (2.22 Å) and **3** (2.10 Å). Infinite framework **3** has a porous sheet structure with hexagonal pores (with *ca.* 18 Å of diameter based on Ag deposition, see the top view in Fig. 2c) that accommodate discrete cages **2** with good shape-complementarity. Interlayer distance of **3** along *c*-axis is 49 Å, which is fitted to the size of packed **2**. All embedded cages **2** in one layer of porous sheets **3** have uniform chirality that arises from the helical orientation of the arms of ligand **1**. The helicity of **1** in the next layer is reversed (as the top and middle layers in Fig. 2a), making co-crystal **2·3** centrosymmetric.‡‡Crystallographic data for **2·3**: *M* = 4741.36, trigonal, space group *P*3_1_*c*, *a* = *b* = 18.1553(5) Å, *c* = 98.0140(10) Å, *V* = 27978.6(16) Å^3^, *Z* = 4, *D*_c_ = 1.126 g cm^−3^, *T* = 100(2) K, 0.818 < *θ* < 19.502°, 8501 unique reflections out of 83 669 with *I* > 2*σ*(*I*), GoF = 1.235, final *R* factors *R*_1_ = 0.0966 (*I* > 2*σ*(*I*)), w*R*_2_ = 0.2921, CCDC deposit number 1999248.

Liquid–liquid diffusion is important for the formation of hybrid structure **2·3**; we obtained only single crystals of the (M_3_L_2_)_6_ hexameric cage (**5** in Fig. 3) from its homogeneous solution.^4^ Presumably, the liquid–liquid diffusion conditions produce a gradient in the local conditions of the mixture in the tube and allow oligomerization of the same M_3_L_2_ subunit into both discrete and infinite frameworks (**2** and **3**, respectively), which in turn co-crystallize into hybrid structure **2·3**. It is interesting that the observed discrete cage is dimeric (M_3_L_2_)_2_ cage **2**, not the hexameric (M_3_L_2_)_6_ cage **5** that was obtained under homogeneous conditions. We suppose that under the liquid–liquid diffusion conditions, kinetically formed dimeric cage **2** is incorporated into co-crystal **2·3** before it can be converted into thermodynamically favourable hexameric cage **5**.

The common M_3_L_2_ subunits in **2** and **3** have similar capped structures, in which the two ligands (outer-**1** and inner-**1**) are associated through weak acetylene–Ag(i) coordination (Fig. S2†). The three arms of outer-**1** in **2** and **3** are almost superimposable. In contrast, those of inner-**1** are oriented quite differently in **2** and **3**. In **2**, the three pyridyl nitrogen atoms are oriented such that the two subunits converge into the discrete structure, whereas those in **3** are directed at different angles to generate the extended coordination network (see Fig. 2b and c). This conformational adaptability is ascribed to the loose acetylene–Ag(i) coordination. In fact, there is a considerable difference in the geometry around the Ag(i) centres in **2** and **3** (average ∠N1⋯Ag1⋯N2 bend angle is 120° for **2** and 151° for **3**; N1 and N2 are the pyridyl nitrogen atoms of outer-**1** and inner-**1**, respectively).

**Fig. 4:**
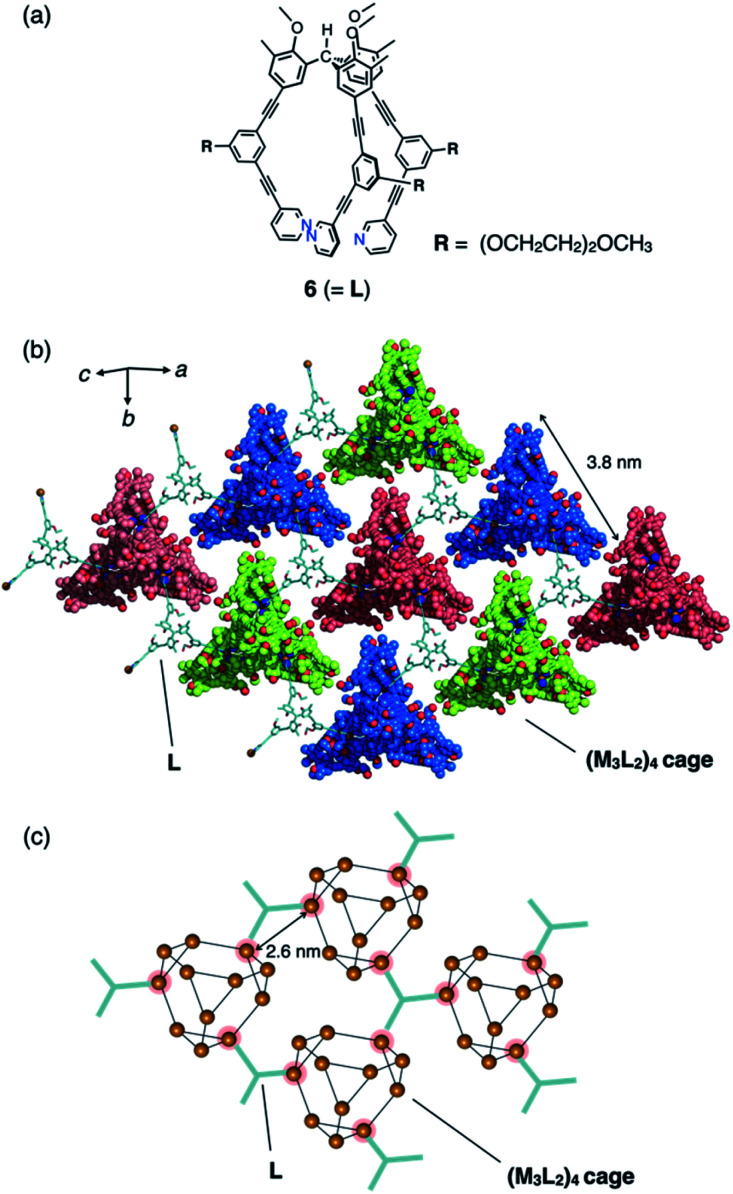
(a) Chemical structure of tripodal ligand **6**. (b) Crystal structure of infinite coordination network **6·7**, in which discrete (M_3_L_2_)_4_ cages **7** are connected into a network. (c) Cartoon representation of network **6·7** showing the twelve Cu(i) centres in the discrete (M_3_L_2_)_4_ cage extracted from the crystal structure.

Another type of discrete–infinite hybridization was achieved when similar tripodal ligand **6** (2.6 mM in nitromethane) with an (OCH_2_CH_2_)_2_OCH_3_ side chain (Fig. 4a) was complexed with CuBF_4_ (3.8 mM), followed by slow vapour-diffusion crystallization with diethyl ether as a poor solvent. After the incubation at 3 °C for 2 weeks, the single crystals were obtained in moderate yield (61%). The framework of resulting discrete (M_3_L_2_)_4_ tetrahedral cage **7** (with *ca.* 3.8 nm of each side length, analogous to cage **4** in Fig. 3; see also Fig. S3 and S4†) was revealed by single-crystal X-ray analysis. This discrete cage is co-crystallized with additional molecules of **6** that cross-link between cages of **7** by coordinating to one Cu(i) center in three neighbouring cages forming two-dimensional network (with intermetallic distances of 2.6 nm, Fig. 4b and c), exhibiting the composition of [(Cu_3_(**6**)_2_)_4_(BF_4_)_12_](**6**)(solv)_*n*_. Considering the crystal packing of **6·7**, geared configuration of two molecules of **6** (Fig. S4†) contributes to the cross-link, which is supported by dipole–dipole interactions and hydrogen bonding among alkoxy moieties of the introduced side chains and methoxy groups of the triarylmethane core. Consequently, two face-to-face networks are woven forming a bilayer (with thickness of 32 Å along *c*-axis) with the uniform chirality of the cage **7**.§§Crystallographic data for **6·7**: *M* = 22030.43, monoclinic, space group *C*2/*c*, *a* = 67.657(10), *b* = 38.962(10) Å, *c* = 67.631(10) Å, *V* = 168 089(56) Å^3^, *Z* = 4, *D*_c_ = 0.862 g cm^−3^, *T* = 293(2) K, 1.724 < *θ* < 47.974°, 45 656 unique reflections out of 156 947 with *I* > 2*σ*(*I*), GoF = 1.562, final *R* factors *R*_1_ = 0.1723 (*I* > 2*σ*(*I*)), w*R*_2_ = 0.4172, CCDC deposit number 1999249.

The cross-linked Cu(i) centres in **6** show tetrahedral geometry with a weakly π-coordinated acetylene moiety. The average ∠N1⋯Cu1⋯N2 bend angle of 99° is considerably smaller than that of the tri-coordinated Cu(i) centres (108°: Fig. 4c). This large deviation in the bend angle demonstrates the structural flexibility and wide scope of the acetylene π-coordination. In network **6·7**, five BF_4_^−^ ions are encapsulated in the cavity of cage **7** (Fig. S5†); this is also observed in discrete capsule **4**.^4^

## Conclusions

In summary, we have synthesized discrete–infinite hybrid structures in which discrete cages with pre-determined cavities are included within infinite frameworks. Recent studies in the field of metal–organic frameworks (MOFs)^7^ and other infinite crystalline materials^8^ emphasize the importance of cavity design in porous coordination networks. We believe that embedding molecular hosts (with predetermined cavities as developed for host–guest chemistry and molecular recognition in solution)^9^ into infinite frameworks (with predetermined lattices) is a promising route to the construction of a diverse structures and properties of cavities in solid materials. The co-crystallization between discrete coordination cages and networked structures reported here is a unique example of this approach. Finally, we pay special attention to the loose acetylene–metal π-coordination because it allowed us to generate both the discrete and infinite frameworks from the same building unit at the same time and thus form the discrete–infinite co-crystal in a single step.

## Conflicts of interest

There are no conflicts to declare.

## Supplementary Material

SC-011-D0SC03191G-s001

SC-011-D0SC03191G-s002
