# Supplementary material for: “Eggs in egg cartons”: co-crystallization to embed molecular cages into crystalline lattices
Source: Chem Sci. 2020 Sep 15;11(38):10457–60. doi: 10.1039/d0sc03191g (PMC8162400; doi:10.1039/d0sc03191g)
Supplement: SC-011-D0SC03191G-s001 [file SC-011-D0SC03191G-s001.pdf]

Electronic Supplementary Material (ESI) for Chemical Science.
This journal is © The Royal Society of Chemistry 2020

Electronic Supplementary Information for

"Eggs in Egg Cartons": Co-crystallization to Embed Molecular Cages into Crystalline Lattices

Yuya Domoto,* Masahiro Abe, Kidai Yamamoto, Takashi Kikuchi and Makoto Fujita*

Contents

1. Experimental procedure…………………………………………………………………2
2. Supplementary Figures……………………………………………………………….…5
3. NMR data…………………………………………………..……………………….8
4. Crystallographic analysis……………………………………………………………10
5. References.…………………………………………………………………………..…12

1. Experimental procedure

1.1 General information

NMR spectra were measured on a Bruker Avance III HD (500 MHz) spectrometer equipped with a PABBO probe and Bruker AV-500 (500 MHz) spectrometer equipped with a CP-TCI cryoprobe. Melting points were determined on an MPA100 OptiMelt (Stanford Research System) and a Yanaco MP-500V apparatus. High-resolution ESI-MS data were recorded on a Bruker maXis spectrometer (Instrument Center at Institute for Molecular Sciences, Okazaki, Japan). Silica gel chromatography was carried out using Silicagel 60N (Kanto Chemical, neutral, spherical) or Biotage SNAP Ultra cartridge. Solvents and reagents were purchased from TCI, FUJIFILM WAKO Pure Chemical Industries, Sigma-Aldrich (Merck), Combi-Blocks and Kanto Chemical. Unless otherwise noted all of the chemicals were reagent grade and used without any further purification.

1.2 Synthesis of ligand 1 and 6

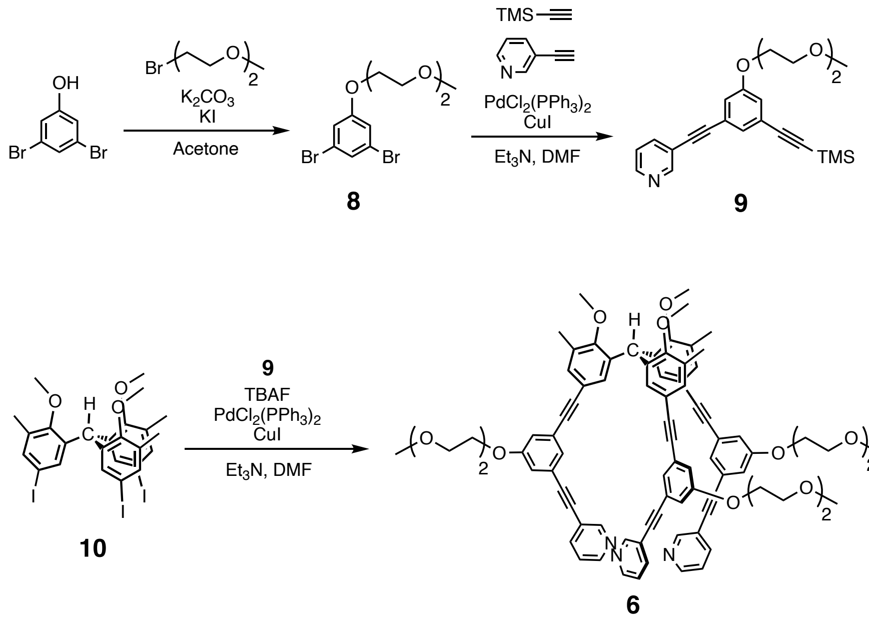

Ligand **1** was prepared according to the previous report[S1].

Synthesis of 1-(2-(2-methylethoxy)ethoxy)-3,5-dibromobenzene 8

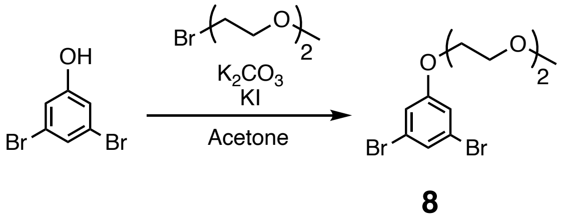

Under argon atmosphere, an acetone (40 mL) solution of 3,5-dibromophenol (3.00 g, 11.9 mmol), K_2CO_3 (8.20 g, 59.3 mmol), KI (0.798 g, 4.81 mmol) and 2-(2-methylethoxy)ethyl bromide (1.59 mL, 11.9 mmol) was stirred under reflux for 24 h. After the solvent was removed, the residue was extracted with dichloromethane and washed with brine. The organic layer was dried over Na_2SO_4 and concentrated under reduced pressure. The residue was purified by silica gel column chromatography (hexane; hexane/ethyl

acetate 9:1 to 4:1) to give 1-(2-(2-methylethoxy)ethoxy)-3,5-dibromobenzene **8**[S2] (4.10 g, 97%) as pale yellow oil.

^{1}H NMR (500 MHz, $CDCl_3$, 300 K): δ 7.25 (s, 1H), 7.02 (d, *J* = 1.5 Hz, 2H), 4.11 (t, *J* = 4.6 Hz, 2H), 3.83 (t, *J* = 4.6 Hz, 2H), 3.71-3.69 (m, 2H), 3.58-3.56 (m, 2H), 3.39 (s, 3H).

Synthesis of arylethynylpyridine 9

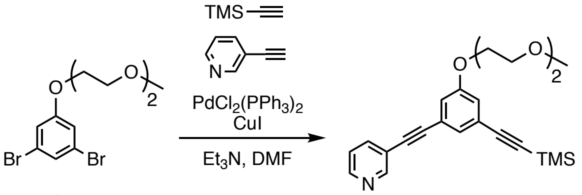

8 **9**

A solution of 1-(2-(2-methylethoxy)ethoxy)-3,5-dibromobenzene **8** (3.54 g, 10.0 mmol) and 3-ethynylpyridine (1.03 g, 9.99 mmol) in DMF (48 mL) was deaerated in vacuo. Then bis(triphenylphosphine)palladium(II) dichloride (353 mg, 0.503 mmol), copper(I) iodide (162 mg, 0.853 mmol), and trimethylsilylacetylene (1.38 mL, 9.98 mmol) were sequentially added. After an addition of triethylamine (48 mL; deaerated by argon bubbling), the mixture was stirred at 60 ºC overnight. After evaporation, the mixture was extracted with ethyl acetate and washed with water and brine. The organic layer was dried over Na_2SO_4 and concentrated by evaporation in vacuo. The crude product was purified by silica gel column chromatography (hexane/ethyl acetate 2:1 to 1:1) to give arylethynylpyridine **9** (1.06 g, 27%) as pale orange oil.

^{1}H NMR (500 MHz, $CDCl_3$, 300 K): δ 8.75 (d, *J* = 1.0 Hz, 1H), 8.55 (dd, *J* = 4.8, 0.8 Hz, 1H), 7.79 (dt, *J* = 7.9, 1.7 Hz, 1H), 7.27-7.30 (m, 2H, overlap with the residual solvent peak), 7.05 (m, 1H), 7.02 (s, 1H), 4.15 (t, *J* = 5.0 Hz, 2H), 3.86 (t, *J* = 5.0 Hz, 2H), 3.71-3.73 (m, 2H), 3.57-3.59 (m, 2H), 3.40 (s, 3H), 0.25 (s, 9H); ^{13}C NMR (126 MHz, $CDCl_3$, 300 K): δ 158.40 (C), 152.30 (CH), 148.75 (CH), 138.47 (CH), 128.00 (CH), 124.51 (C), 123.62 (C), 123.06 (CH), 120.16 (C), 118.53 (CH), 118.29 (CH), 103.84 (C), 95.03 (C), 91.69 (C), 86.20 (C), 71.97 (CH_2), 70.84 (CH_2), 69.60 (CH_2), 67.79 (CH_2), 59.11 (CH_3), −0.12 (CH_3); HR-ESI-TOF-MS: *m*/*z* calcd for $C_{23}H_{27}NO_3NaSi$: 416.1658 ([M+Na]$^+$); found: 416.1660.

Synthesis of ligand 6

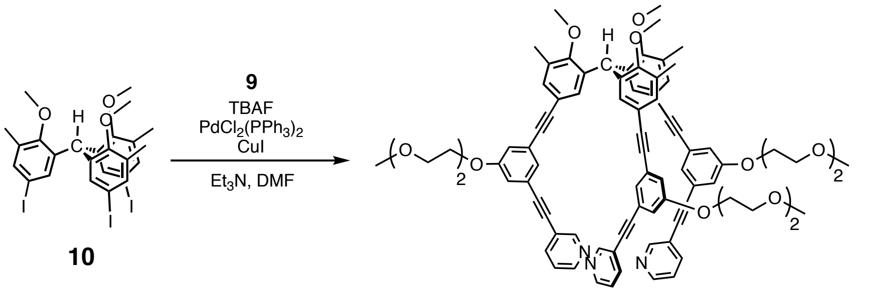

10 **6**

A solution of triiodide **10**[S3] (298 mg, 0.395 mmol), protected acetylene **9** (613 mg, 1.56 mmol) in DMF (10 mL) was deaerated in vacuo. Then bis(triphenylphosphine)palladium(II) dichloride (27.6 mg, 0.0393 mmol), copper(I) iodide (13.2 mg, 0.0693 mmol), triethylamine (18 mL; deaerated by argon bubbling), and tetrabutylammonium fluoride (1 M THF solution, 2.0 mL) were sequentially added. The mixture was stirred at room temperature for 30 min and then stirred at 70 ºC overnight. After concentration under reduced pressure, the mixture was diluted with water and ethyl acetate. The extracted organic layer was washed with brine. Then the solution was dried over Na_2SO_4 and concentrated by evaporation in vacuo. The crude product was purified by silica gel column chromatography (chloroform/methanol 20:1 to 10:1) and GPC (chloroform) in a low to give ligand **6** (257 mg, 50 %) as a pale yellow solid.

Mp 65.9-67.2 ºC; ^{1}H NMR (500 MHz, $CDCl_3$, 300 K): δ 8.72 (brs, 3H), 8.53 (brs, 3H), 7.75 (brd, J = 7.5 Hz, 3H), 7.26-7.32 (m, 12H), 7.03-7.04 (m, 6H), 6.92 (brs, 3H), 6.53 (s, 1H), 4.14 (brm, 6H), 3.83 (brm, 6H), 3.68 (brm, 6H), 3.54-3.59 (m, 15H), 3.36 (s, 9H), 2.32 (s, 9H); ^{13}C NMR (126 MHz, $CDCl_3$, 300 K): δ 158.49 (C), 157.07 (C), 152.26 (CH), 148.68 (CH), 138.49 (CH), 136.92 (C), 134.00 (CH), 131.67 (C), 131.05 (CH), 127.51 (CH), 124.72 (C), 123.66 (C), 123.06 (CH), 120.20 (C), 118.11 (C), 117.98 (CH), 117.94 (CH), 91.81 (C), 90.24 (C), 87.70 (C), 86.18 (C), 71.94 (CH_2), 70.79 (CH_2), 69.58 (CH_2), 67.79 (CH_2), 60.15 (CH_3), 59.08 (CH_3), 37.66 (CH), 16.38 (CH_3); HR-ESI-TOF-MS: m/z calcd for $C_{85}H_{79}N_3O_{12}$: 1334.5737 ($[M+H]^+$); found: 1334.5829.

2. Supplementary Figures

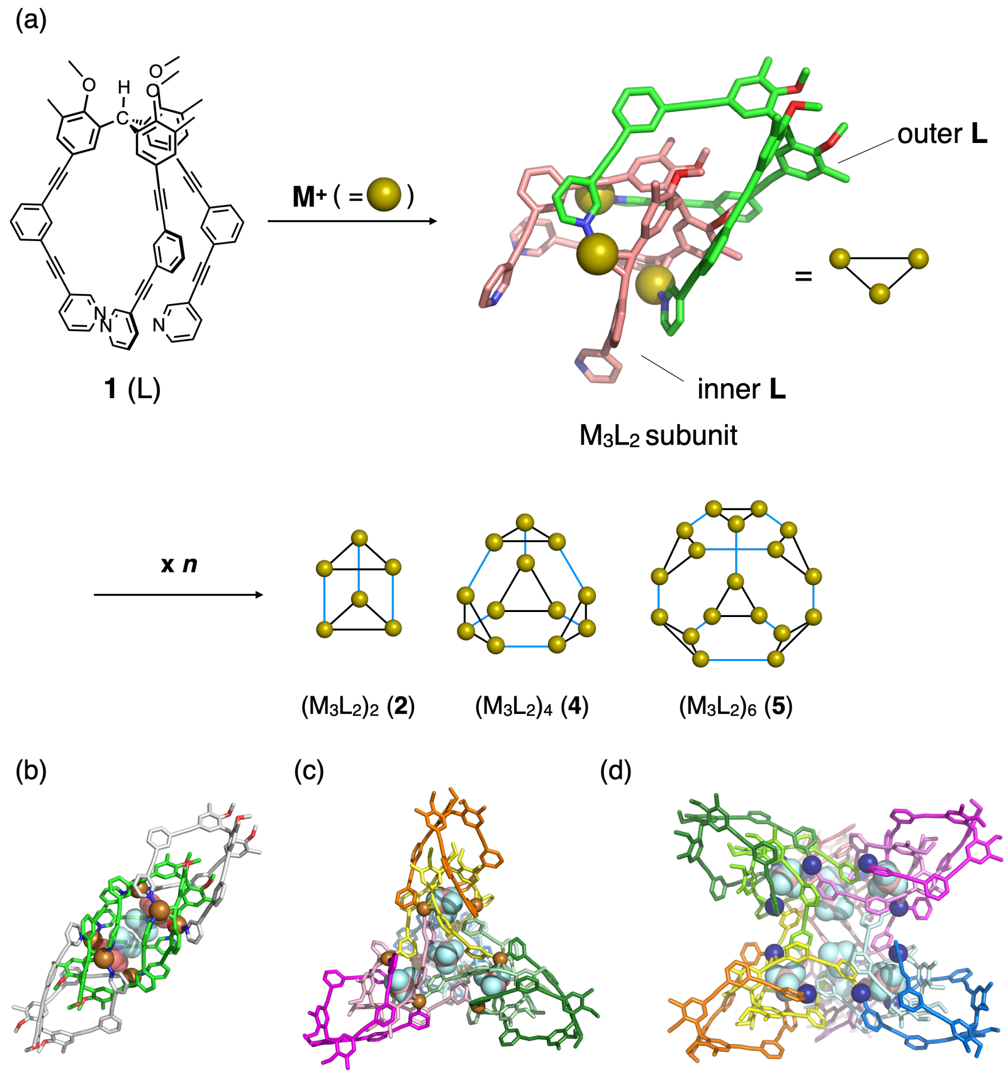

Figure S1. Our previously reported self-assembly of coordination polyhedra via oligomerization of M_3L_2 subunits powered by concerted metal-acetylene interaction and pyridyl coordination at work. (a) A scheme for the formation of a hypothetical M_3L_2 subunit and its subsequent oligomerization to give $(M_3L_2)_n$ coordination cages **2** ($n = 2$), **4** ($n = 4$), and **5** ($n = 6$). (b) Crystal structure of cage **2** (M = Cu(I); encapsulated two $CF_3SO_3^-$ ions are presented as CPK model). (c) Crystal structure of cage **4** (M = Cu(I); with encapsulated five BF_4^- ions). (d) Crystal structure of cage **5** (M = Ag(I); with encapsulated eight BF_4^- ions). For details, see our previous report[S1].

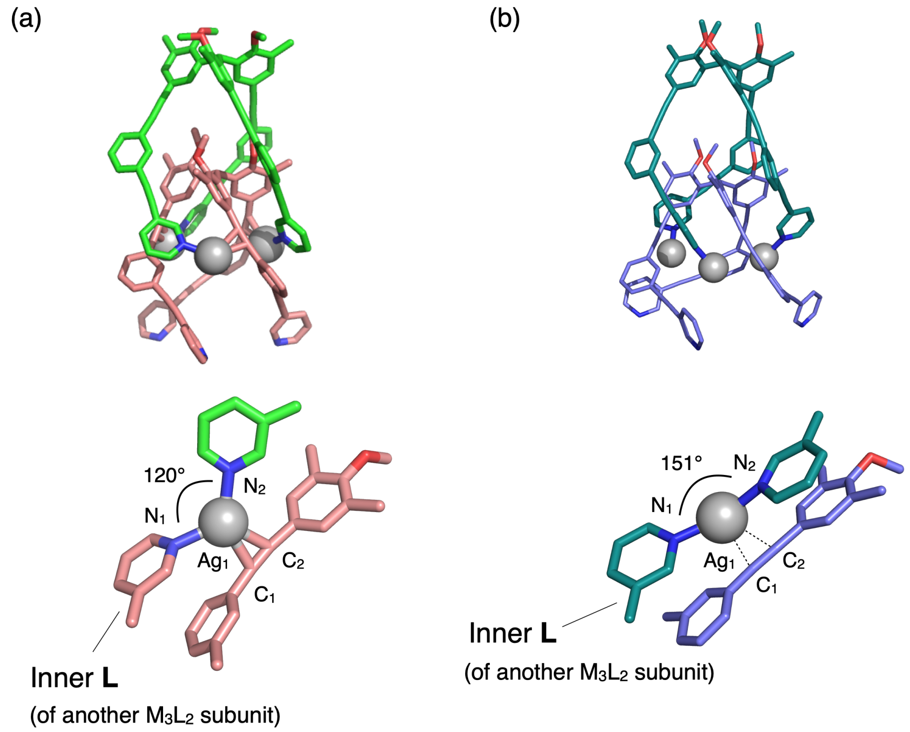

Figure S2. Partial views of the X-ray structure of the discrete-infinite co-crystal **2•3**. (a) Coordination center of the discrete cage **2**. (b) Coordination center of the infinite complex **3**. For **3**, average distance between Ag(I) and acetylene carbons is 2.93 Å, which is less than the sum of VDW radii of Ag (1.72 Å) and C (1.70 Å).

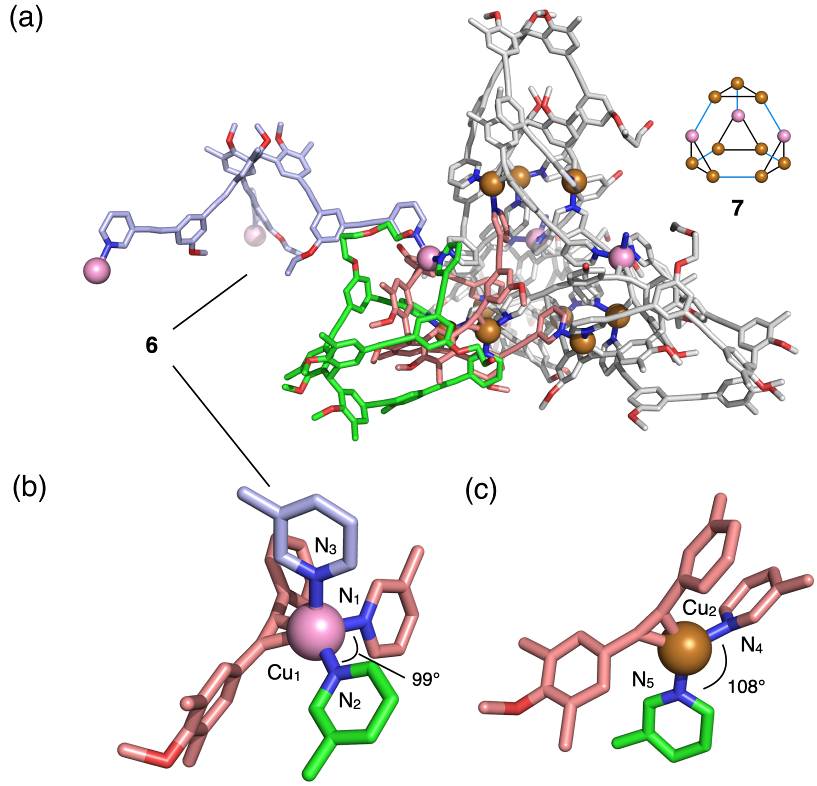

Figure S3. Crystal structure of infinite coordination network **6•7.** For clarity, bridged tetracoordinated copper atoms are colored as pink: (a) Partial crystal structure of $(M_3L_2)_4$ cage **7** bridged by the ligand **6** (one of four M_3L_2 subunits in the framework of **7** was colored), (b) A bridged tetracoordinated center of **7**, (c) A non-bridged tricoordinated center of **7**.

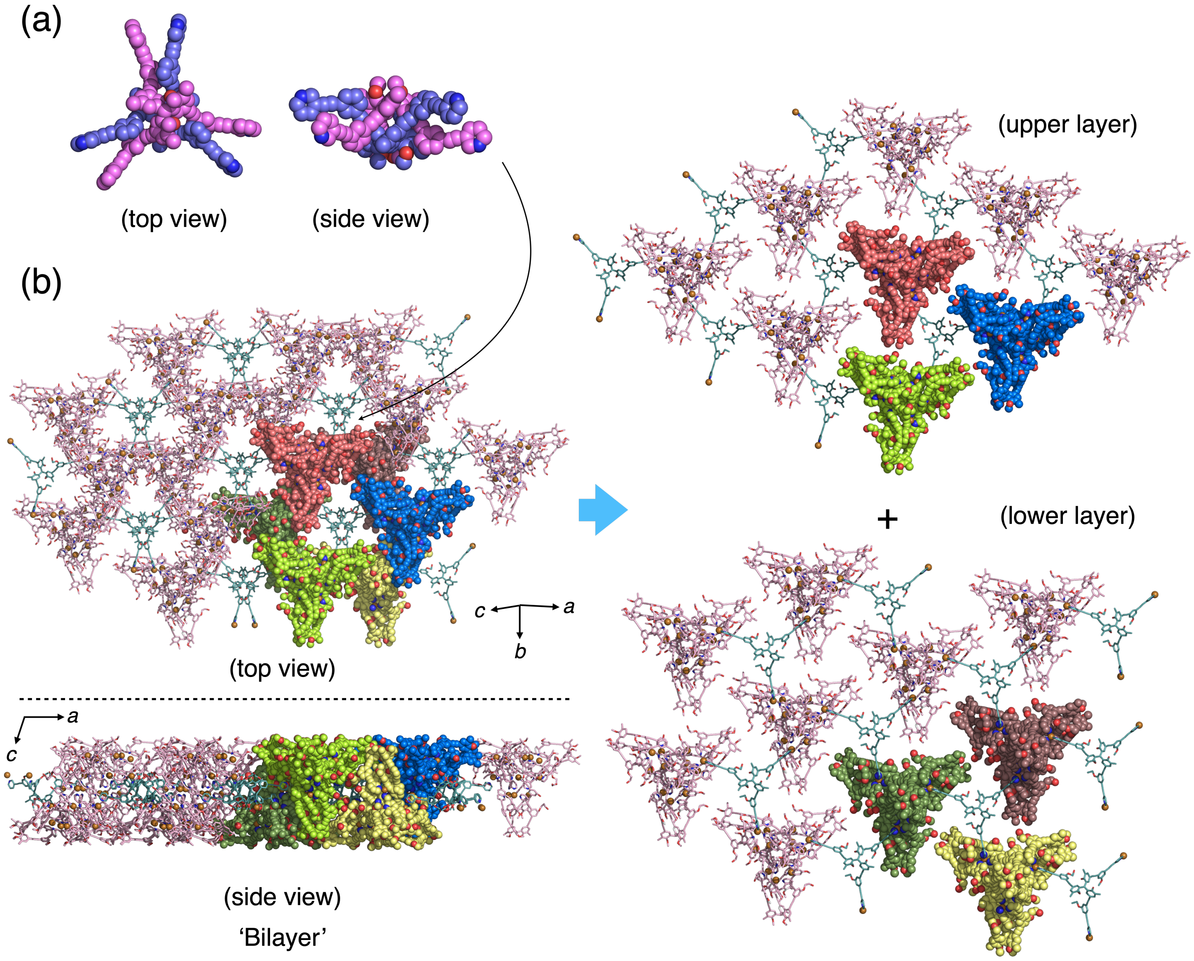

Figure S4. Packing structure of **6•7**. (a) Two molecules of ligand **6** gearing to form a pair. Here, one ligand **6** (colored as magenta) coordinates to three cages of **7** in the lower layer, while the other (blue) coordinates to those in the upper layer. (b) As described in the main article, ligand **6** cross-links the cage **7** giving 2D sheet-like complex. Note that two *face-to-face* sheet structures are interlocked as a result of the cross-linking style shown in (a). Among this 'bilayer' structure, helical chirality of cage **7** is same, while that of the neighboring layer is opposite to afford the centrosymmetric crystal packing.

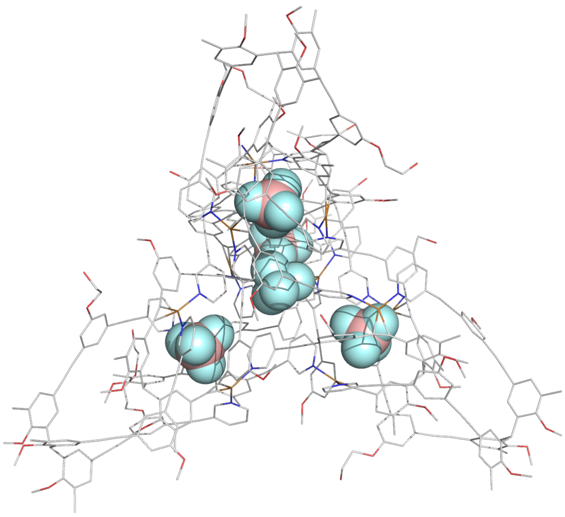

Figure S5. Encapsulated BF_4^- ions (CPK model) in the cage **6** in the crystal structure of **6•7**.

3. NMR data

3.1 NMR data of synthesized compounds

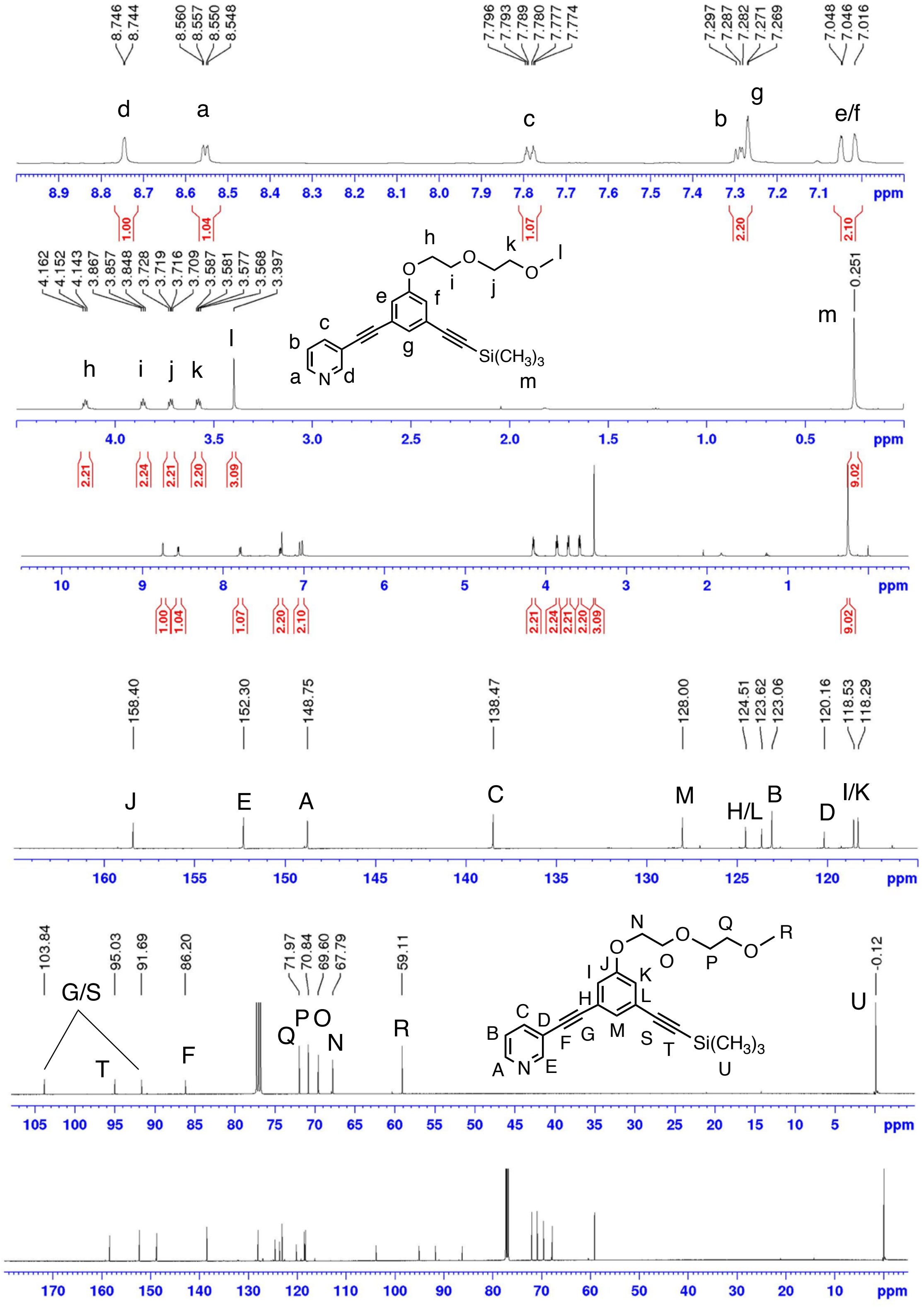

Figure S6. ^{1}H and ^{13}C NMR spectra of arylethynylpyridine **9** (500/126 MHz, $CDCl_3$, 300 K).

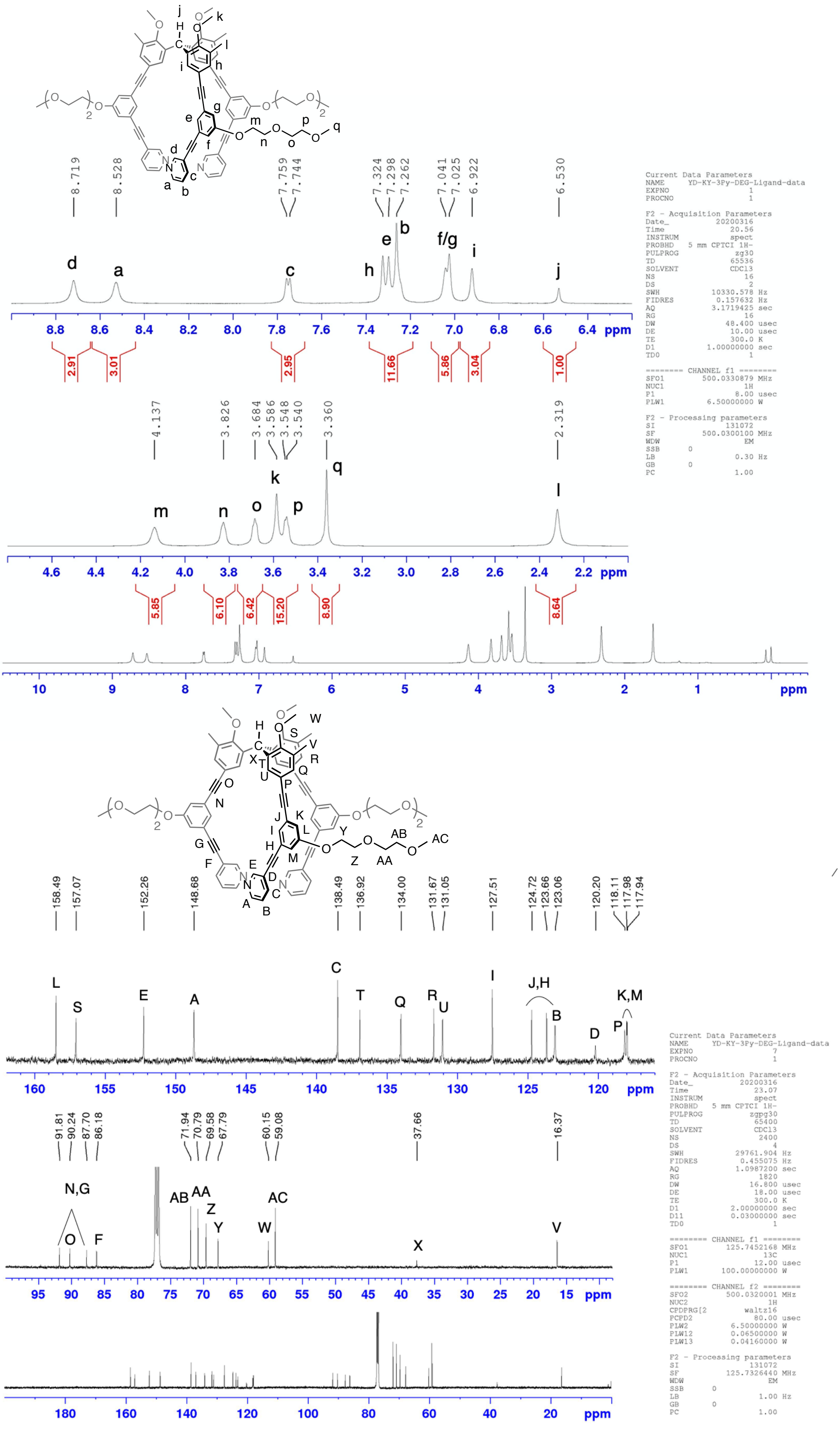

Figure S7. ^{1}H and ^{13}C NMR spectra of ligand **6** (500/126 MHz, $CDCl_3$, 300 K)

4. Crystallographic analysis

Single crystals of the co-crystal **2•3** were prepared by liquid-liquid diffusion method. Namely, a solution of $AgBF_4$ (7.9 mM, 150 μL) in MeOH was carefully deposited on a solution of ligand **1** (5.2 mM, 150 μL, $CHCl_3$) covered by $CHCl_3$/MeOH (50/50, v/v) buffer in advance, in a capped microtube. Incubation at 303 K for two weeks gave the crystals (y. 56%). The diffraction data were collected at BL41XU (EIGER X 16 M detector) at SPring-8. The crystals were removed from the microtubes and picked up by the cryo-loop (Hampton), and mounted on a goniometer. Data collection was performed at 100 K.

Single crystals of the co-crystal **6•7** was prepared by slow vapour diffusion of diethyl ether into a CD_3NO_2 solution prepared from ligand **6** (2.6 mM in nitromethane) and $[Cu(MeCN)_4]BF_4$ (3.75 mM) in a glass tube at 276 K. Substantial amount of crystals was obtained after 2 weeks (y. 61%). The crystals were removed from the microtubes and introduced into a glass capillary (borosilicate, Hampton Research), and mounted on a goniometer. The diffraction data were collected at the BL26B1 beamline (EIGER4M (DECTRIS) detector) at the Spring-8. Data collection was performed at 293 K. Diethyleneglycol side chains were severely disordered, thus only the initial several atoms could be assigned. Some of BF_4^- ions were also disordered. Phase purity of the crystalline materials was confirmed by screening of the X-ray irradiation for (1) several crystals obtained from the same crystallization tube, and (2) different parts on the same crystal with concentrated beam-size, giving identical cell parameters to those finally determined by the main diffraction measurements.

XDS[S4] was used for the processing and data reduction. The structures were solved and refined by SHELXT[S5] and SHELXL.[S6] Detailed crystallographic data are summarized in Table S1. PyMOL 2.0 (Schrödinger, LLC) was used for the production of graphics.

Table S1. Crystal data and structure refinement for co-crystal **2•3** and **6•7**.

	**2•3**	**6•7**
Identification code	MA-080-12_a_P-31c	KY-047-3deg-crystal02
CCDC number	1999248	1999249
Empirical formula	$C_{280}H_{196}Ag_6B_2F_8N_{12}O_{12}$	$C_{1328}H_{819}B_{10.3}Cu_{24}F_{41.2}N_{54}O_{130}$
Formula weight	4741.36	22030.43
Temperature	100(2) K	293(2) K
Wavelength	0.700 Å	1.000 Å
Crystal system	Trigonal	Monoclinic
Space group	$P3_1c$	$C2/c$
Unit cell dimension	$a = b$ = 18.1553(5) Å,<br>c = 98.0140(10) Å<br><br>$\alpha = \beta$ = 90°,<br>γ= 120°	a =67.657(10) Å,<br>b = 38.962(10)Å,<br>c = 67.631(10) Å<br><br>$\alpha = \gamma$ = 90°,<br>β =109.4650(10)°
Volume	27978.6(16) $Å^3$	168089(56) $Å^3$
Z	4	4
Density (calculated)	1.126 g/cm^3	0.862 g/cm^3
Absorption coefficient	0.454 mm^{-1}	0.869 mm^{-1}
$F(000)$	9680	45293
θ range for data collection	0.818 to 19.502°	1.724 to 47.974°
Index ranges	$-17 \leqq h \leqq 17$<br>$-17 \leqq k \leqq 17$<br>$-91 \leqq l \leqq 93$	$-54 \leqq h \leqq 54$<br>$-30 \leqq k \leqq 30$<br>$-54 \leqq l \leqq 54$
Reflection collected	83669	156947
Independent reflections	8501 [R_{int} = 0.0737]	45656 [R_{int} = 0.1240]
Data / restrains / parameters	8501 / 85 / 945	45656 / 12197 / 7052
Goodness-of-fit on F^2	1.235	1.562
Final R indices [I>2σ(I)]	R_1 = 0.0966<br>wR_2 = 0.2921	R_1 = 0.1723<br>wR_2 = 0.4172
R indices (all data)	R_1 = 0.1166<br>wR_2 = 0.3173	R_1 = 0.2584<br>wR_2 = 0.4699
Largest diff. peak and hole	1.20 and −0.77 e $Å^{-3}$	0.98 and −0.56 e $Å^{-3}$

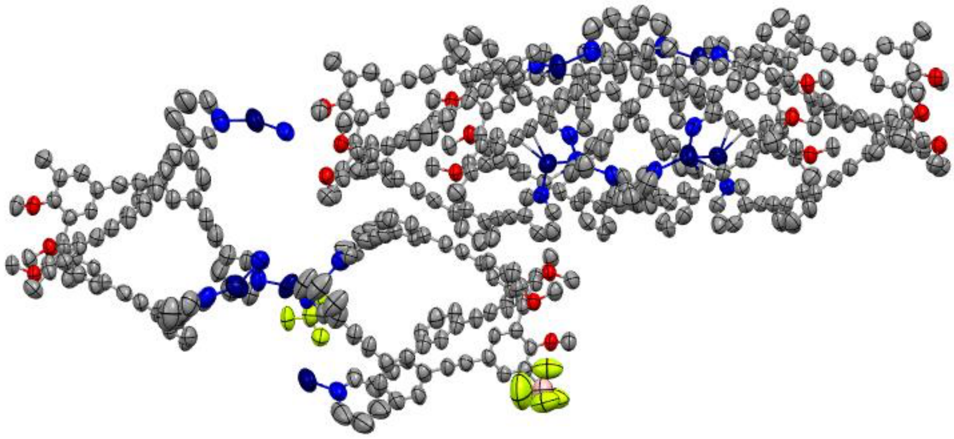

Figure S8. ORTEP drawing (50% probability ellipsoids) of the co-crystal **2•3**.

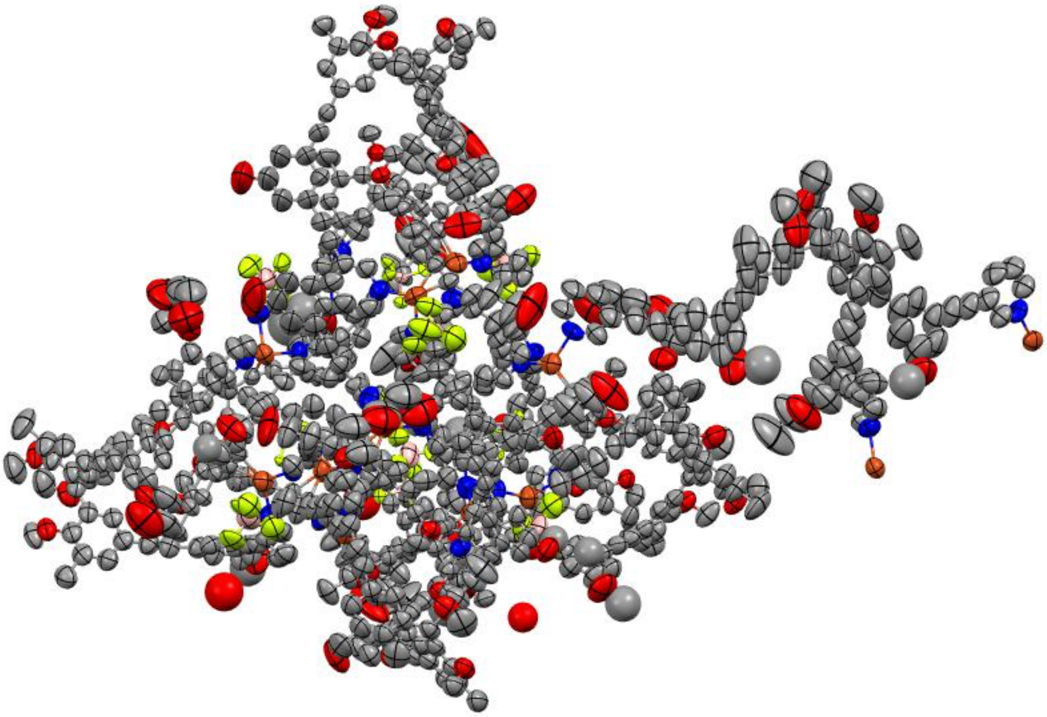

Figure S9. Crystal ORTEP drawing (50% probability ellipsoids) of **6•7** (except for severely disordered diethyleneglycol side chains partially modeled isotropically).

5. Supplementary References

[S1] Y. Domoto, M. Abe, T. Kikuchi, M. Fujita, *Angew. Chem. Int. Ed.* **2020**, *59*, 3450-3454.

[S2] Y. Wang, F. Bie, H. Jiang, *Org. Lett.* **2010**, *12*, 3630-3633.

[S3] J. Kobayashi, Y. Domoto, T. Kawashima, *Chem. Commun.* **2009**, 6186-6188.

[S4] W. Kabsch, *Acta Crystallogr. D* **2010**, *66*, 125-132.

[S5] G. M. Sheldrick, *Acta Crystallogr. A* **2015**, *71*, 3-8.

[S6] G. M. Sheldrick, *Acta crystallogr. C* **2015**, *71*, 3-8.
